# Type VI Secretion System in Pathogenic *Escherichia coli*: Structure, Role in Virulence, and Acquisition

**DOI:** 10.3389/fmicb.2019.01965

**Published:** 2019-08-30

**Authors:** Fernando Navarro-Garcia, Fernando Ruiz-Perez, Ángel Cataldi, Mariano Larzábal

**Affiliations:** ^1^Department of Cell Biology, Centro de Investigación y de Estudios Avanzados del IPN (CINVESTAV-IPN), Mexico City, Mexico; ^2^Department of Pediatrics, University of Virginia School of Medicine, Charlottesville, VA, United States; ^3^Laboratorio de Escherichia coli, Instituto de Agrobiotecnología y Biología Molecular (IABIMO) INTA-CONICET, Buenos Aires, Argentina

**Keywords:** diarrheagenic *E. coli*, type 6 secretion system, genome plasticity, protein translocation, effector proteins, bacterial competition, inner and outer membranes

## Abstract

Bacterial pathogens utilize a myriad of mechanisms to invade mammalian hosts, damage tissue sites, and evade the immune system. One essential strategy of Gram-negative bacteria is the secretion of virulence factors through both inner and outer membranes to reach a potential target. Most secretion systems are harbored in mobile elements including transposons, plasmids, pathogenicity islands, and phages, and *Escherichia coli* is one of the more versatile bacteria adopting this genetic information by horizontal gene transfer. Additionally, *E. coli* is a bacterial species with members of the commensal intestinal microbiota and pathogens associated with numerous types of infections such as intestinal, urinary, and systemic in humans and other animals. T6SS cluster plasticity suggests evolutionarily divergent systems were acquired horizontally. T6SS is a secretion nanomachine that is extended through the bacterial double membrane; from this apparatus, substrates are conveyed straight from the cytoplasm of the bacterium into a target cell or to the extracellular space. This nanomachine consists of three main complexes: proteins in the inner membrane that are T4SS component-like, the baseplate complex, and the tail complex, which are formed by components evolutionarily related to contractile bacteriophage tails. Advances in the T6SS understanding include the functional and structural characterization of at least 13 subunits (so-called core components), which are thought to comprise the minimal apparatus. So far, the main role of T6SS is on bacterial competition by using it to kill neighboring non-immune bacteria for which antibacterial proteins are secreted directly into the periplasm of the bacterial target after cell–cell contact. Interestingly, a few T6SSs have been associated directly to pathogenesis, e.g., roles in biofilm formation and macrophage survival. Here, we focus on the advances on T6SS from the perspective of *E. coli* pathotypes with emphasis in the secretion apparatus architecture, the mechanisms of pathogenicity of effector proteins, and the events of lateral gene transfer that led to its spread.

## Introduction

**FIGURE 1:**
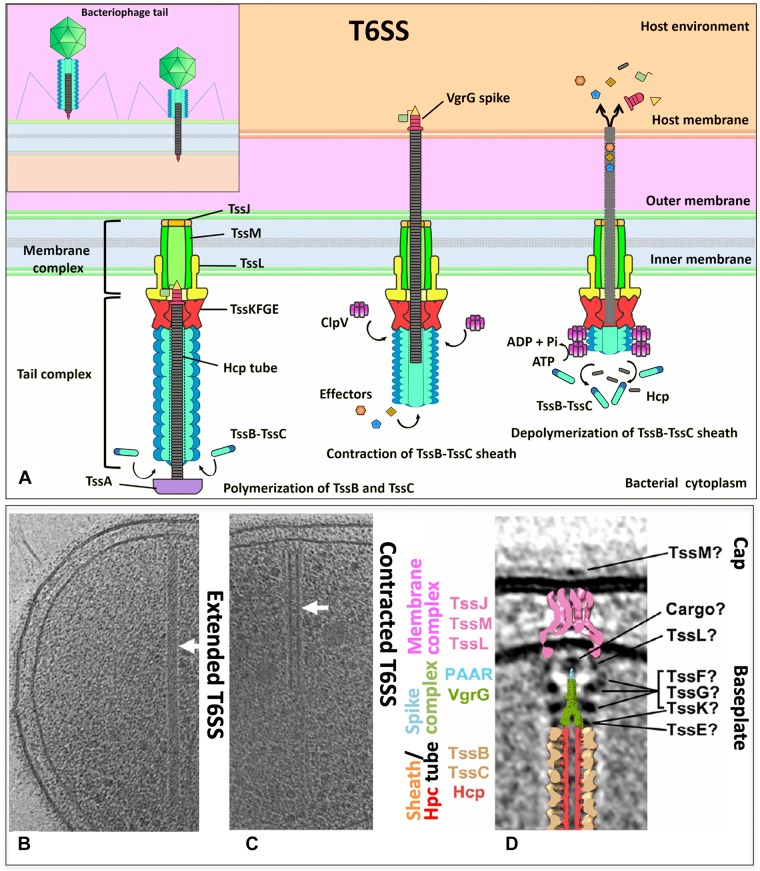
**(A)** The T6SS is composed of a membrane complex, a baseplate and a tail complex. TssJ–TssL–TssM makes the membrane complex and is connected to the TssB–TssC tail sheath and the hemolysin co-regulated protein (Hcp) inner tube through the baseplate (composed of TssK, TssE, and VgrG). Effectors are recruited to the spike–tube complex through the extension domains of VgrG and/or PAAR-repeat proteins and through incorporation into Hcp tube. An unknown extracellular signal triggers sheath contraction, which leads to the ejection of the spike–tube complex across the target membrane, thereby delivering effector proteins into the cell. The ATPase ClpV disassembles the contracted TssB–TssC sheath, which enables a new T6SS complex to be reassembled from the released subunits. **(B,C)** Tomographic slices through an extended T6SS **(B)** and a contracted T6SS **(C)** (arrows) in frozen-hydrated *Myxococcus xanthus* cells. **(D)** Central slice of the sub-tomogram average of *M. xanthus* T6SS membrane-associated region and placing the central slices of available structures. **(B–D)** Modified from Chang et al., EMBO reports e201744072. Copyright© 2017 by John Wiley Sons, Inc. Reprinted by permission of John Wiley & Sons, Inc.

The T6SS (type VI secretion system) is one of a recent specialized secretion system identified in Gram-negative bacteria (Figure 1A). T6SS gene clusters are widely distributed in proteobacteria and may exist in several chromosomal copies (Bingle et al., 2008; Journet and Cascales, 2016). Initially, the T6SS was associated with bacterial virulence concerning eukaryotic host cells, but a scarce number of T6SSs are directly implicated in cell disruption. For instance, *Vibrio cholerae* delivers toxin modules that interfere with the host cytoskeleton (Pukatzki et al., 2007). *Pseudomonas aeruginosa* harbors a serine–threonine protein kinase (STPK) that contributes to virulence in neutropenic mice (Wang et al., 1998). *P. aeruginosa* invades epithelial cells by delivering the VgrG2b effector by a T6SS, which interferes with a multiprotein complex catalyzing microtubule nucleation (Sana et al., 2015). In *Legionella pneumophila*, the chromosomal cluster *icmGCDJBF* is required for the killing of human macrophages (Purcell and Shuman, 1998). In *Salmonella enterica*, a genomic island [*Salmonella* centrisome island (SCI)] related to the *icmF* gene cluster is associated to eukaryotic cells invasion (Folkesson et al., 2002). In *Salmonella* Enteritidis strain P125109, a gene SEN1005 downstream of a trimmed T6SS region plays a role in altering the expression of genes involved in the invasion of bacteria into non-phagocytic cells as well as in bacterial engulfment by macrophages and acute inflammation in C57BL/6 mice (Silva et al., 2012; Das et al., 2018). Still, the main T6SS role appears to be related to bacterial competition through killing neighboring bacteria without cognate immunity protein by the secretion of proteins with antibacterial activity directly into the periplasm of the target bacteria after cell–cell contact. Most pathogens harboring a T6SS are an important threat to the human health, including several biowarfare agents of category A or B, i.e., *Yersinia pestis*, *V. cholerae*, *Burkholderia mallei*, *Francisella tularensis*, pathogenic *Escherichia coli*, *Salmonella typhimurium*, and emerging and opportunistic pathogens including *Burkholderia cenocepacia*, *P. aeruginosa*, *Edwardsiella tarda*, and *Aeromonas hydrophila* (Folkesson et al., 2002; Das and Chaudhuri, 2003; Rao et al., 2004; Dudley et al., 2006; de Bruin et al., 2007; Mougous et al., 2007; Schell et al., 2007; Shalom et al., 2007; Zheng and Leung, 2007; Aubert et al., 2008; Suarez et al., 2008; Yen et al., 2008).

## Structure of the T6S Apparatus

The type VI secretion apparatus is formed by a double-membrane-spanning structure that, like many other secretion systems, work by the one-step mechanism where bacterial cytoplasmic substrates are conveyed directly into a target cell or to the extracellular space (Figure 1A). The partially/totally folded substrates are transported as in the chaperone–usher pathway (Costa et al., 2015). The minimal apparatus components called core (Table 1) are thought to be formed by 13 subunits (Bingle et al., 2008; Boyer et al., 2009), and in numerous cases, the core components also contain additional proteins (Cascales and Cambillau, 2012). Although the functions of many additional proteins are unknown, it is thought they might modulate or facilitate the T6SS assembly or allow supplementary functions to this nanomachine (Boyer et al., 2009; Schwarz et al., 2010). Prevalent clustered genes that encode components of a T6SS belong to proteobacterial genomes averaging over 20 kb (Lesic et al., 2009; Schwarz et al., 2010; Gode-Potratz and McCarter, 2011). These multiplied systems do not seem redundant due to their sequence divergence, different phenotypic profiles, and divergent regulation. Instead, it has been suggested that due to the T6SS cluster plasticity in high taxonomic ranks, these systems with evolutionarily divergence could be acquired horizontally. Indeed, five different phylogenetic groups harbor T6SS gene clusters (Bingle et al., 2008; Boyer et al., 2009).

**TABLE 1:** The T6SS core components of EHEC O157:H7 strain EDL933.

**Name**	**ORFs**	**COG**	**Localization**	**Function**
TssJ	Z0257	COG3521	OM	Membrane complex
TssL	Z0255	COG3455	IM	Membrane complex
TssM	Z0250	COG3523	IM	Membrane complex
TssK	Z0256	COG3522	IM	Baseplate complex
TssF	Z0249	COG3515	IM	Baseplate complex
TssG	Z0259	COG3520	IM	Baseplate complex
TssE	Z0261	COG3518	CP	Baseplate complex
TssA	Z0251	COG3515	CP	Tail complex
TssB	Z0264	COG3157	CP	Tail complex (sheath)
TssC	Z0262	COG3517	CP	Tail complex (sheath)
ClpV	Z0254	COG0542	CP	ATPase
Hcp	Z0248	COG3157	CP/IM	Tail complex (Hcp tube)
VgrG	Z0267	COG3501	IM	Spike

*OM, outer membrane; IM, inner membrane; CP, cytoplasmic; ORF, open reading frame; COG, clusters of orthologous groups of proteins.*

T6SS nanomachinery is formed by three major complexes: the membrane complex, the tail complex, and the baseplate complex (Table 1); the first is located in the inner membrane (IM) and proteins resemble components of the T4SS (Pukatzki et al., 2006; Ma et al., 2009), the tail complex is formed by components evolutionarily related to the phage’s contractile tails (Leiman et al., 2009), while the baseplate is a protein complex used for the tail assemblies serving as a platform during assembly of the tube and sheath but also triggers the contraction of the sheath (Cherrak et al., 2018).

### IM Complex

The full T6SS assembly is formed by the trans-envelope membrane complex that tethers the baseplate onto which the tail polymerizes (Figure 1A). Unlike the baseplate, tube, and sheath proteins, which are conserved among contractile injection systems, the membrane complex is specific to the T6SS (Durand et al., 2015). Besides the anchor of the baseplate to the IM, the membrane complex also functions as a channel to permit the passage of the tail tube-spike and for maintaining the attacking cell integrity throughout the inner tube translocation (Durand et al., 2015). The minimal T6SS membrane complex is composed of the TssJ, TssL, and TssM proteins (Zoued et al., 2014). The IM protein TssM connects the outer membrane (OM) lipoprotein TssJ to the IM protein TssL (Ma et al., 2009) (Figures 1A,D). The TssJLM complex from enteroaggregative *E. coli* (EAEC) analyzed by negative-stain electron microscopy (EM) showed that 10 TssJ lipoproteins are bound to 10 TssM proteins, constructing a double concentric rings of arches and pillars throughout the periplasm. A flexible base composed of the TssM N-terminal part and 10 TssL copies is linked by the arches, while the TssJLM complex is assembled into a trans-envelope structure of fivefold symmetric by rotation (Durand et al., 2015). TssM is a key component of the membrane complex as a connector of the IM and OM. The cytoplasmic domain of TssM allows the TssM oligomerization and interacts with itself and with TssL (which also has a cytoplasmic domain that interacts with TssK and TssE) and with the baseplate components TssK and TssG (Logger et al., 2016; Zoued et al., 2016). TssK is localized in the cytoplasm, and this cytoplasmic subunit connects the whole membrane complex (TssJ–TssL–TssM) to the tail components. TssK is a T6SS baseplate component (Zoued et al., 2013) (Figure 1D). The crystal structure of the full-length TssK protein from EAEC shows a trimeric structure whose N-terminal domain shares an unexpected homology with the siphophage receptor-binding protein (RBP) shoulders (Nguyen et al., 2017). The N-terminal domain of TssK is attached to the rest of the baseplate, while the C-terminal domain is bound to the T6SS membrane complex and uses it as a receptor to dock the baseplate. The N-terminal shoulder and the C-terminal head are globular domains and are separated by the helical stalk. The TssK C-terminal domain flexibility could establish a flexible link to keep the anchorage of the baseplate to the membrane complex along the whole process of contraction of the tail (Nguyen et al., 2017).

### Tail Complex

The T6SS membrane complex attaches the tail complex proteins to the IM and OM. While the TssB–TssC complex forms the tail sheath, which is perpendicular both to the membrane and a long tubular structure, it is deeply extended into the bacterial cytoplasm (Basler et al., 2012). An inner tube is inside the tail sheath, which is formed by polymers of Hcp (hemolysin co-regulated protein) (Leiman et al., 2009) (Figures 1B,C). The inner tube forms a tube of stacked Hcp hexamers *in vitro* and has been shown essential to the T6SS function. The hexameric rings of 80–90 Å wide formed by the six Hcp molecules are assembled and stabilized by an extended intersubunit belt. These hexamers are assembled as tubes of 35- to 40-Å inner diameter, accommodating therefore a small protein in a partly folded or folded state. Hcp is assembled in a tubular structure from the IM and passes by the OM since *in vivo* studies have shown Hcp is accumulated in the culture supernatant and in the periplasm. However, it is unknown how the Hcp assembly is controlled since the Hcp packing manner *in vivo* has not been detected yet (Cascales and Cambillau, 2012). Constitution into the *E. coli* heterologous host (lacking other T6SS components) and *in vitro* experiments showed that a direct interaction occurs between the Hcp tube component and the VgrG (valine–glycine repeat G) spike. The N-terminal domain of VgrG was required to interact with Hcp, to start the correct Hcp tube polymerization, as well as to promote sheath dynamics and Hcp release (Renault et al., 2018). The VgrG trimer is located centrally in the baseplate complex forming a spike. This spike appears to be a platform of nucleation for assembling the tail tube of the T6SS (Leiman et al., 2009; Basler et al., 2012).

TssB and TssC are needed for assembling the sheath (Figure 1D) in a similar way as in the bacteriophage T4 tail, where the contractile sheath formed by gp18 subunits engulfs the inner tube (Figure 1A, insert). In all T6SS gene clusters, these two genes (*tssB* and *tssC*) co-occur, and encoded proteins interact and stabilize each other (de Bruin et al., 2007; Bröms et al., 2009; Bönemann et al., 2009; Aubert et al., 2010; Lin et al., 2013; Lossi et al., 2013; Zhang et al., 2013). The N-terminus of TssC contacts TssB, and this region is needed for the interaction of TssB and TssC (Aubert et al., 2010; Zhang et al., 2013), as well as a conserved α-helix in the TssB central region where the hydrophobic face is involved in the TssB–TssC interaction (Bröms et al., 2009; Zhang et al., 2013). Structural data on the EAEC TssB C-terminal domain have been reported (Douzi et al., 2018). Purified TssB protein multimerize to form trimers in solution, and the first 86 amino acids of the N-terminal region govern this multimerization. TssB undergoes proteolytic cleavages to accumulate two fragments, and the C-terminal shorter fragment TssB(87–165) includes a long helix (termed H1) and a bundle of smaller helices (termed H2 and H3). H1 residues (highly conserved) are involved in the interaction with TssC, and the H2–H3 hairpins (conformed by several charged residues, highly variable) are critical for sheath assembly and T6SS function (they may mediate contacts with the baseplate) (Douzi et al., 2018). TssB–TssC heterodimers appear to polymerize to build the tail sheath around the growing Hcp tube of an external diameter of 300 Å and an internal diameter of 100 Å and hundreds of angstroms long (Bönemann et al., 2009). The structure shows four β-strands assembled in a core domain provided by one TssB and two TssC proteins to stabilize the sheath. The contraction mechanism is still not clearly comprehended since there is no structure at high resolution of the T6SS sheath in its extended form. However, it is possible to detect these extended and contracted conformations (Chang et al., 2017) (Figures 1B,C). The tubule formed by TssB–TssC contracts to push the Hcp tube to the cell exterior previous to secretion as the bacteriophage T4 sheath. The ClpV ATPase is thought to provoke this contraction through depolymerization of TssB–TssC tubules (Bönemann et al., 2009). ClpV is a cytosolic protein belonging to the family of Hsp100 AAA + proteins, which are required for substrate unfolding by acting as hexameric ATPases. The chaperone of ClpV (TssH) might act during T6SS assembly and function by first causing the cytosolic TssB–TssC tubules depolymerization, permitting their transport to the periplasm. This process leads to the TssB–TssC polymerization to form the sheath structure again. Second, ClpV provides energy required for its contraction through depolymerizing the sheath (Bönemann et al., 2009). ClpV is specific for a given T6SS gene cluster as different ClpV from different clusters are not interchangeable. This specificity is originated by the binding of N-terminal domains of ClpV and that of their cognate TssC1 proteins (Douzi et al., 2016).

### Baseplate Complex

**FIGURE 2:**
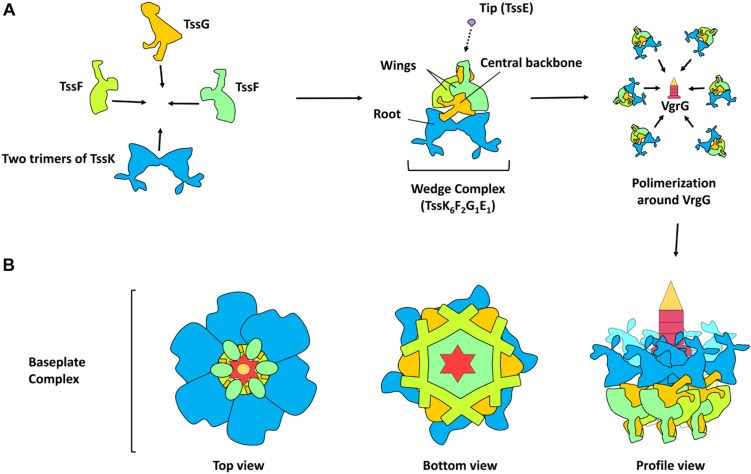
Model of T6SS baseplate assembly. **(A)** Schematic representation of the protein-protein interaction in the assembly of the TssKFGE wedge complex. The wedge complex is composed of five essential subunits: TssK, TssF, TssG, TssE with a stoichiometry of 6 (two trimers), 2, 1 and 1 subunits, respectively (theoretical mass, 498 KDa). According to the Cryo-EM density map, two wing-like structures (TssF) are wrapping a central backbone (TssG and probably TssE) and a root-like structure (TssK). Biogenesis of the baseplate occurs by the polymerization of six wedges around the central VgrG. **(B)** Schematic representation of the baseplate assembly complex (top, bottom and profile view).

Unlike the understanding of T6SS nanomachine assembly and function, the T6SS baseplate composition, which would be similar to the bacteriophage baseplate, had remained until very recently unknown. Initially, it was found that TssAFGK are essential core components of the baseplate. TssK is a cytoplasmic trimeric protein associated with the IM-forming oligomers of higher order (Casabona et al., 2013; Zoued et al., 2013; English et al., 2014). Also initially, isolation of the complex showed that a complex between VgrG and TssF–TssG–TssE can be isolated and that an interaction network among several components of the baseplate and tail components is required, including TssE, TssG, TssF, TssA, TssK, and VgrG (Figure 1D). All these six T6SS proteins are required for assembling the tail tube. Furthermore, for the baseplate recruitment to the final T6SS apparatus, the initial formation of the membrane complex including the interaction of TssG in the baseplate and the IM TssM protein is needed (Brunet et al., 2015). This baseplate is an assembly platform to build the tube and sheath. For the assembly of these structures, the baseplate is recruited to the IM complex (mentioned above) formed by the OM lipoprotein TssJ and the two IM proteins TssL and TssM. These interactions occur through multiple contacts such as TssG and TssK with the TssM cytoplasmic loop and TssK with the TssL cytoplasmic domain (Zoued et al., 2013; Brunet et al., 2015; Logger et al., 2016). The TssL cytoplasmic domain of EAEC also interacts with another subunit of the baseplate (TssE) through the L6–L7 loop. The interaction between the cytoplasmic domain of TssL and TssK is mediated by the disordered L3–L4 loop, while the conserved groove of the TssL cytoplasmic domain binds the TssM cytoplasmic loop in the IM (Zoued et al., 2016). The T6SS baseplate is assembled through intermediates, TssFG, TssKFG, and TssKFGE, and these have structural and functional homologies to the bacteriophage wedges (Cherrak et al., 2018). Recently, two independent groups have shown data on the stoichiometry, architecture, and role of baseplate complex in the T6SS apparatus of EAEC (Cherrak et al., 2018; Park et al., 2018). A cryo-EM reconstruction (assembled from TssK, TssF, and TssG) at 3.7-Å resolution of an EAEC baseplate subcomplex showed two TssK trimers interacting with a symmetrical complex formed by two copies of TssF and one of TssG (Cherrak et al., 2018; Park et al., 2018). TssKFGE wedges could polymerize around the VgrG hub (Figure 2) to form a hexagonal baseplate (Cherrak et al., 2018). Hexamerization of the (TssK)6–(TssF)2–(TssG)1–(TssE)1 wedge leads to formation of the baseplate complex around a VgrG trimer bound to a PAAR-repeat protein at its distal extremity (Park et al., 2018). Detailed information on the molecular organization of the TssKFGE baseplate wedge complex (Figure 2), from the model organism EAEC, showed the fully assembled T6SS baseplate is 337 Å in diameter and 180 Å in height (Cherrak et al., 2018). Thus, the TssKFG complex and TssE form a wedge, and then six wedges are assembled circularly in a baseplate formed by several copies of TssK (36), TssF (12), TssG (6), and, probably, six of TssE (Park et al., 2018). In the case of the T6SS baseplate interaction with the tail sheath, the ring of the TssFGE wedge binds to the sheath structure and the main interactions occur between the D2 domain of one TssF (TssFb) and the N-terminal antenna of TssBC (Cherrak et al., 2018). On the other hand, for the connection between the T6SS baseplate and membrane complex, the main contacts are the following: TssK binds to both TssL and TssM (Zoued et al., 2016; Nguyen et al., 2017), TssL binds to TssE, and TssM binds to TssG (Brunet et al., 2015; Zoued et al., 2016). Interestingly, these later interactions were not found in the structure recently reported, probably reflecting a dynamic assembly process. However, the TssK connector ring position corroborates that TssK is the main factor for mediating the docking of baseplate to the membrane complex (Cherrak et al., 2018). Two separated evidence lines showed that TssK trimer orientation in the baseplate positions the TssK_S_ domains (a domain that shares homology with siphophage RBPs) in interaction with the TssFG cap complex, while the TssK_H_ domains (a specific C-terminal head domain) extend in a different direction contrary to the sheath where the membrane complex would be located (Nguyen et al., 2017; Cherrak et al., 2018). It is worthy to mention that, after the VgrG-nucleated assembly of individual baseplate wedges, the baseplate is recruited to the membrane complex (TssJLM) earlier than the contractile tail structure assembly (Brunet et al., 2015; Park et al., 2018). Finally, the T6SS baseplate is the tail assembly platform and also docks the tail to the membrane complex and consequently functions as an evolutionary adaptor (Cherrak et al., 2018).

The T6SS TssE protein and the component gp25 of the bacteriophage baseplate share homology, and the former is needed to TssB/TssC assembly (Basler et al., 2012; Brunet et al., 2013; Kapitein et al., 2013). The gp25 protein might initiate the polymerization of the sheath in the bacteriophage T4 tail by a mechanism of arm exchange using the first row of gp18 subunits (Leiman and Shneider, 2012). Despite the fact that T6SS sheaths do not form in a *tssE* mutant (Basler et al., 2012; Brunet et al., 2013; Kapitein et al., 2013), its precise role in T6SS biogenesis is still unknown. A key component during T6SS biogenesis in EAEC is the TssA protein (Zoued et al., 2017). Recently, it has been proposed that TssA participates in numerous stages of the pathway of T6SS assembly by binding to the membrane complex, perhaps acting as a chaperone for controlling its assembly. TssA helps several functions such as baseplate recruitment and priming and coordination of the extension of tail tube/sheath, as well as to maintain the sheath in an extended conformation. Ultimately, TssA connects the tube distal end and the sheath, allowing the proper tube/spike propulsion when the last row of sheath is contracted (Zoued et al., 2017). Recently, using novel approaches, the identification of the closeness partners *in vivo* of the TssA protein in EAEC was reported. TssA sequentially interacts with different complexes: it engages first with the membrane complex (TssL and TssM) and then with proteins of the baseplate (TssF, TssG, TssK, and VgrG), and, finally, with the tail (Hcp and TssC), confirming previous observations. Furthermore, TssA interacts with TagA in EAEC. However, TagA was not considered a T6SS core component since the presence of the *tagA* gene is associated with a limited number of T6SS gene clusters, and specifically, competition experiments showed EAEC TagA is not essential for T6SS activity. However, TagA plays a role in optimizing T6SS efficiency since the *tagA* mutant retained 12 ± 4% of antibacterial activity versus the wild-type strain (Santin et al., 2018). Indeed, it was recently found that the T6SS proximal (anchored to the baseplate) and distal ends were associated with opposite sides of the cell envelope. Moreover, TssA1 mediates the termination of polymerization and the binding of the distal end through interaction with TssM1 (in the membrane complex) and TagA. Both ends of the EAEC T6SS1 bound to the cell envelope allow bidirectional contractions: T6SS contracts not only toward its proximal end (canonical contraction) but also toward its distal end (non-canonical contraction). The C-terminal domain of TssA1 plays an important role in the processes at the distal end and, along with TagA, might act as an alternative baseplate that can trigger contraction at the distal end (Szwedziak and Pilhofer, 2019).

### Effector Delivery

**FIGURE 3:**
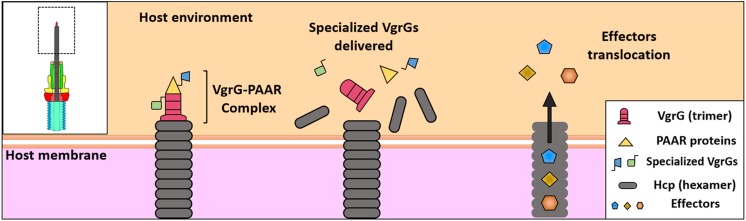
Protein translocation by T6SS. “Specialized” VgrGs effectors are associated with Hcp-VgrG-PAAR complex. The T6SS contraction projects the Hcp tube through the membrane, delivering the effectors into the cytosol of the target cell. Finally, the “cargo” effectors are translocated into the cytosol by the Hcp tube and the spike.

It has been noted the T6SS machinery can translocate effectors in the following ways: (i) bound to structural components as specialized VgrG effectors or (ii) through non-covalent interaction with any of the components of the core (cargo effectors) (Durand et al., 2014; Whitney et al., 2014). Since in these cases, the effector being translocated has to be associated with Hcp–VgrG–PAAR that are components of the expelled structure (Figure 3), it is thought that several effectors associated to this puncturing protein are delivered at once in one lethal shot inside the target cell (Shneider et al., 2013). It is thought this delivery in one lethal shot allows a single event of T6SS sheath contraction (one toxic payload of effectors) by these T6SS. According to this idea and more fascinating, Ho et al. provided direct evidence that a T6SS of *V. cholerae* delivers effector domains, both homologous and heterologous, into the cytosol of the target cell, and they are then trafficked to other subcellular locations such as periplasm through signals carried within these effectors (Ho et al., 2017).

The Hcp tube appears to be assembled on the trimer of VgrG, propelling it inside the target cell impulsed by TssBC contraction as it occurs by analogy with the phage tail. VgrG penetrates the target cell through its needle-shaped β-helical domain. Findings supporting this model include (i) both Hcp and VgrG are delivered into the bacterial supernatant in codependency (Pukatzki et al., 2007; Zheng and Leung, 2007), (ii) Hcp and VgrG interact with each other (Lin et al., 2013), and (iii) the correct assembly of the Hcp tube requires the VgrG protein (Brunet et al., 2014). Bioinformatics analyses have showed that Hcps with C-terminal extension toxins, designated as Hcp-ET, exist widely in Enterobacteriaceae (Table 2). Among them, Hcp-ET1 from Shiga toxin-producing *E. coli* (STEC) showed inhibition of bacterial growth through the predicted HNH-DNase activity, which was T6SS-dependently delivered, while Hcp-ET2 from ETEC was also able to mediate interbacterial antagonism through a Tle1 phospholipase (containing a DUF2235 domain) activity. On the other hand, Hcp-ET3-4 from uropathogenic *E. coli* (UPEC) is fused with two toxin domains in the C-terminus, Pyocin S3 and colicin DNase, and the gene for this protein is followed by three duplications of genes of the cognate immunity protein. Interestingly, this toxin had antibacterial effects, and all duplications of the cognate immunity protein contributed to neutralize the DNase toxicity of Pyocin S3 and colicin. Thus, Hcp-ET proteins are polymorphic T6SS effectors and, thus, present a novel encoding pattern of T6SS effectors (Ma et al., 2017a). Remarkably, several VgrGs harbor extensions in the C-terminal toward the end of the beta-prism that might work as effector domains; thereby, all these VgrGs are called “specialized VgrG” (Pukatzki et al., 2007, 2009). It has been shown that specialized VgrGs containing additional domains are transported into target cells (Ma et al., 2009); thus, VgrG would work as a spike as in bacteriophages. Additionally, PAAR, a protein with a conical structure encoded within *vgrG* gene operons of T6SS, caps VgrG extremity, refining the spike tip (Shneider et al., 2013). Thus, PAAR and VgrG are codependently secreted, and PAAR has been proposed as a connector protein among VgrG and other effector proteins (Figure 3) since putative effector domains are fused to several PAAR proteins (Shneider et al., 2013). Moreover, PAAR function could be more complex since the release of Hcp to the extracellular medium is PAAR dependent, and thereby, PAAR is important for T6SS assembly and function; it has even been proposed that PAAR is needed for nucleating VgrG folding (Shneider et al., 2013).

**TABLE 2:** T6SS effector proteins from *Escherichia coli*.

**Effector**	***E. coli* pathotype**	**Target**	**Effector activity**	**Gene**	**References**
AaiC	EAEC	Unknown (secreted)	Unknown (Hcp homologs)	BQ8769_135	Dudley et al., 2006
Rhs-CT1	STEC	Prokaryotic	Metalloprotease	CRM83_12975	Ma et al., 2017b
Rhs-CT3	ETEC	Prokaryotic	Restriction endonuclease-3	rhsA_2	Ma et al., 2017b
Rhs-CT5	EHEC	Prokaryotic	RNAase (Toxin_55)	Z5014	Ma et al., 2017b
KatN	EHEC	Eukaryotic	Catalase intramacrophage survival	Z1921	Wan et al., 2017
Tle1	EAEC	Prokaryotic/Eukaryotic	Type VI lipase toxin	EC042_4534	Journet and Cascales, 2016
Tle4	APEC	Prokaryotic	Type VI lipase toxin	YP_542179	Ma et al., 2014
Hcp-1	NMEC	Eukaryotic	HBMECs	G8IRM9_ECOLX	Zhou et al., 2012
Hcp1, Hcp3	ExPEC	Eukaryotic	Bacterial–eukaryotic cell interactions	NZ-CP006632.1 (0225 & 4211)	Peng et al., 2016
Hcp2	ExPEC	Prokaryotic	Bacterial competition	NZ-CP006632.1 (0245)	Peng et al., 2016
Hcp-ET1	STEC	Prokaryotic	HNH-DNase	AAF13_24945	Ma et al., 2017a
Hcp-ET2	ETEC	Prokaryotic	Tle1 phospholipase	A31K_02346	Ma et al., 2017a
Hcp-ET3-4	UPEC	Prokaryotic	Pyocin S3 and colicin DNase	UTI89_C0121	Ma et al., 2017a
XmtU	APEC	Prokaryotic	peptidase_C39 toxin	AHW84522	Ma et al., 2017b
VT1	ETEC	Prokaryotic	Amidase	KX118292	Ma et al., 2017a
VT5	ETEC	Prokaryotic	Lysozyme-like	KX118293	Ma et al., 2017a
VgrG1, VgrG2	ExPEC	Prokaryotic/eukaryotic	Antibacterial and virulence phenotypes	PCN033_0247 PCN033_1587	Zong et al., 2019

As the T6SS effectors coexist with their cognate immunity protein, T6SS-dependent effectors have been experimentally found by screening immunity proteins (Dong et al., 2013) and mutating the effector/immunity genes for comparing the secretomes of wild-type strain and mutants (Hood et al., 2010; Altindis et al., 2015). In other cases, bioinformatics identification has been used as in the cases of peptidoglycan-degrading effectors such as Tae (an amidase) and Tge (a glycoside hydrolase) superfamilies (Russell et al., 2012; Whitney et al., 2013). The co-occurrence of effector with cognate immunity protein is directly located within the T6SS clusters or downstream of VgrG (Russell et al., 2012, 2013). The encoding patterns for these effectors are diverse, including from simple proteins with a single domain to large proteins with multidomain (Russell et al., 2012; Brooks et al., 2013). Rhs proteins (formerly thought to be rearrangement hot spot elements) with polymorphic C-terminal toxin domains that inhibit or kill neighboring cells were identified (Jackson et al., 2009; Zhang et al., 2012). All of them encode an N-terminal PAAR motif, which, as mentioned above, sharpens the VgrG trimer spike by forming a conical structure on the tip (Shneider et al., 2013). Ma et al. (2017b) found a novel Rhs in *E. coli* with a domain of metallopeptidase-4 (MPTase4), and this designated Rhs-CT1 showed an antibacterial activity depending on the T6SS. Using the domain architecture of Rhs-CT1 including Rhs with an N-terminal PAAR motif and a C-terminal toxin domain, the authors bioinformatically detected a number of Rhs-CTs initially in *E. coli* (Rhs-C1 to Rhs-C10), but, finally, more than 400 putative Rhs-CT proteins with an N-terminal PAAR motif were found in *E. coli*. Among them, Rhs-CT3 (with a restriction endonuclease-3 domain) and Rhs-CT5 (a RNase) were experimentally confirmed to cause interbacterial antagonism (Ma et al., 2017b). Furthermore, other analyses showed *vgrG*_*O*__1_ and *eagR*/DUF1795 (an effector-associated gene for Rhs that interact with VgrG) located upstream of *rhs-ct* are needed for the delivery of Rhs-CTs, perhaps as a T6SS chaperone. Additional to chaperoned Rhs-CTs, neighborless Rhs-CTs that have been classified into a distinct family (Rhs-Nb) exist, which share an evolutionary relationship with T6SS2-Rhs (coded in the *E. coli* into the T6SS2 cluster). By extending this bioinformatics approach, approximately 2,500 N-terminal PAAR motifs of Rhs-CTs were found in 143 bacterial species, and according to the predicted toxic activity, they were divided into eight clans: DNase, RNase, deaminase, endonuclease, peptidase, pore forming, protein modifying, and unknown encoding (Ma et al., 2017b).

## T6SS in *E. coli*

Several T6SSs have been found in many sequenced strains from pathogenic *E. coli.* There are strains that harbor more than one T6SS and/or strains with a single T6SS cluster but containing multiple copies of *hcp* and *vgrG* genes. Most research on T6SS in *E. coli* has been carried out in EAEC and APEC (avian pathogenic *E. coli*) pathotypes. It has been reported that in EAEC strain 17-2, two gene clusters (called Sci-1 and Sci-2) are harbored in the *pheU* pathogenicity island (PAI), encoding subunits of two different T6SS (Dudley et al., 2006). EAEC is a pathogenic *E. coli* pathotype that may cause persistent and severe diarrhea in children, infants, and young children as well as immunocompromised individuals (Harrington et al., 2006; Estrada-Garcia and Navarro-Garcia, 2012). On the other hand, three different T6SS were found in APEC collections with a frequency of 14.6% for T6SS1, 2.3% for T6SS2, and 0.8% for T6SS3. Notably, 85% of APEC strains harboring T6SSs loci were identified in virulent phylogenetic groups D and B2 in an analysis of the *E. coli* genomes (Johnson et al., 2008), strongly suggesting that T6SSs contribute to the pathogenicity of this pathotype (Ma et al., 2013). In fact, in the APEC strain SEPT362, Hcp and IcmF proteins reduce adherence in the interaction of bacteria with endothelial cells and induce actin cytoskeleton rearrangements showing that T6SS influences the expression of type 1 fimbria and pathogenicity of this strain (de Pace et al., 2010, 2011). More interestingly, the APEC K1 strain TW-XM harbors two T6SS: the first (T6SS1) is multifunctional since it has a role in the bacteria–host cell interaction, formation of biofilm, and bacterial competition; while the second (T6SS2) is able to influence the bacterial interaction with the mouse brain microvascular endothelial cell line in an exclusive and significant way (Ma et al., 2014).

Recently, it was found that *E. coli* strains with extensive drug resistance (XDR) properties that also harbor different secretion systems including the T6SS. *E. coli* strain Sanji (from a pheasant colibacillosis outbreak) harbors a total of 32 genes of antibiotic resistance revealed by whole-genome sequencing, most of them associated with IS26 (insertion sequence 26) elements. This Sanji strain was classified in the sequence type ST167 group, and further comparative genome sequence analysis with other 14 ST167 strains revealed ST167 genomes are highly similar; some strains contain up to 12 distinct resistance genes. Three insertions found in Sanji and other ST167 strains harbor a T3SS, a T2SS, and a T6SS (a 30.6-kb insertion). Moreover, the Sanji strain exhibited growth inhibition against *E. coli* TOP10 (a laboratory strain) (Zeng et al., 2019). Thus, the concurrence of antibiotic resistance genes and T6SS could cause the next XDR superbug to emerge as it occurred with recent O104:H4 clonal lineages, which acquired not only Shiga toxin-encoding phage but also ESBL resistance (Boisen et al., 2015).

Genome analysis of porcine ExPEC strain PCN033 shows a T6SS that encodes three distinct *hcp* gene families. Proteins from the Hcp family are associated with competition between bacteria and in infections to other cells. Thus, these proteins have diverse functions, but these three Hcp are involved in mice colonization; Hcp2 mostly functions in bacterial competition, while Hcp1 and Hcp3 mainly contribute to bacterial–eukaryotic cell interactions (Peng et al., 2016).

By using proteomic and microarray analyses, it was identified that the master regulator of virulence AggR in EAEC activates several chromosomal genes, among them 25 neighboring T6SS-encoding genes designated *aaiA* to *Y*, harbored in the PheU PAI (Dudley et al., 2006). Two secreted effector proteins designated *aaiC* were identified at that time, which currently are known to be Hcp homologs. To identify essential components in AaiC biogenesis, authors constructed EAEC strain mutants in *aaiB*, *aaiG*, *aaiO*, or *aaiP*, which did not affect AaiC expression but affected its secretion. Furthermore, it was shown the operon composed of *aaiA-P* is sufficient for AaiC secretion in *E. coli* HB101 (Dudley et al., 2006).

Most T6SS targets in prey bacteria are the cell wall peptidoglycan and membrane phospholipids (Table 2). However, a variety of other targets have been described recently. For instance, induction of *soxS* in *E. coli* is the result of T6SS lethal attacks, similar to the effect of P1vir phage and polymyxin B. Dong et al. (2015) detected enhanced reactive oxygen species (ROS) levels with a fluorescent probe correlating with *soxS* induction in *E. coli*. The role of T6SS in other *E. coli* pathotypes is not well known; however, the presence of T6SS operons has been reported in many other *E. coli* pathotypes (Petty et al., 2010; Richards et al., 2015). T6SS in these pathogens may probably have similar effector functions as those reported in EAEC. However, T6SS in pathogens with an invasive and intracellular lifestyle may have additional functions.

### Protein Translocation Into Prokaryotic and Eukaryotic Cells

In recent years, several T6SS effectors have been found and characterized, including eukaryotic cell-targeting and antibacterial effectors (Table 2). Thus, a wide range of delivered effectors exist by T6SS, which highlights the variety of activities and cell kind of targets related to this secretion apparatus. These features of T6SS also highlight questions yet under investigation, such as how these effectors are recognized for being secreted and how they are secreted. T6SS effectors are either associated within the Hcp lumen or directly or indirectly bound to the VgrG or PAAR spike as was explained above (Figure 3).

Interestingly, a porcine ExPEC strain PCN033 containing a functional T6SS showed to harbor four *vgrG* genes: two putative *vgrG*s located inside the T6SS cluster (*vgrG1* and 0248), and the other two outside this cluster (*vgrG2* and 1588). However, only the VgrG1 protein is involved in the antibacterial ability and also in the interaction with eukaryotic cells *in vitro*. Furthermore, deletion of *vgrG1*, but not the other *vgrG*s, caused a decrease in the multiplication capacity of PCN033 in an animal model. Remarkably, VgrG1 and VgrG2 are 99% homologous (with 541 amino acids), and 028 and 1588 are identical (with 158 amino acids); the latter two have no gp27/gp5 domain. Moreover, although only VgrG1 exhibited the phenotypes, VgrG2 is different in one amino acid, and trans complementation of either VgrG1 or VgrG2 in the Δ*vgrG1*Δ0248Δ*vgrG2*Δ1588 mutant restored both the antibacterial and virulence phenotypes. These data suggest that a little expression of endogenous VgrG2 is overcome by overexpression on the plasmid (Zong et al., 2019).

In the case of Hcp, there are four distinct Hcp types in Gram-negative bacteria, and three are widely distributed in APEC. Interestingly, transcription levels are divergent among these three *hcp* clusters in duck serum; *hcp1* is upregulated by releasing Fur repression, and the host serum activates the *hcp2B* operon by H-NS derepression for interbacterial antagonism. Notably, these Hcp proteins exhibit significant differences in their extended loop regions, which are related to their functional properties; the loop L2–L3 of variant region Vs2 in Hcp1 and Hcp2B is crucial for the delivery (Tle4 and XmtU, respectively) of antibacterial effectors and the inhibition of macrophage phagocytosis. Thus, these Hcp homologs are functionally different due to differences in transcriptional regulation, extended loop regions, and effector delivery (Ma et al., 2018b).

### Effectors for Targeting Eukaryotic Cells

As mentioned before, effector proteins can be delivered by specifically associating to the Hcp, VgrG, or PAAR protein, covalently or non-covalently as “specialized” or “cargo” effectors, respectively (Cianfanelli et al., 2016). The N-terminal region of *E. coli* CFT073 VgrG (c3393) has been crystalized, and it forms a trimer, but unlike the structurally similar T4 gp27–gp5 complex, VgrG does not harbor the lysozyme domain (Leiman et al., 2009). Additionally, it has also reported that VgrG from *E. coli* O157 (VgrG1) and *E. coli* CFT073 (c3393) are also trimers, and the full-length VgrGs contain similar secondary structure to the T4 gp27–gp5 complex and the lysozyme domain is also absent (Uchida et al., 2014).

In several Gram-negative bacteria have been shown the induction of T6SS loci in infection models or using eukaryotic cell lines; in fact, loss of a functional T6SS impairs bacterial pathogenicity for the host (Hachani et al., 2016). Even when, some of these phenotypes have been reported for *E. coli*, the effectors associated to these observations remains yet elusive.

A bioinformatic analysis showed that in the genome of the meningitis-causing *E. coli* K1 strain RS218, a cluster encoding a putative T6SS exists. A gene cluster deletion mutant (from *evfB* to *hcp1*) was impaired in binding to and invasion of human brain microvascular endothelial cells (HBMECs) compared with the wild-type strain. Furthermore, Hcp1 and Hcp2, which were localized in the bacterial OM, differentially interacted with HBMECs. *hcp2* mutant was deficient in the bacterial binding to and invasion of HBMECs, while Hcp1 induced actin cytoskeleton rearrangement, apoptosis, and interleukin-6 (IL-6) and IL-8 release in HBMECs (Zhou et al., 2012). A similar result was previously found in the APEC strain SEPT362, in which mutants of T6SS core genes (*clpV* and *hcp*) showed decreased adherence and actin rearrangement on epithelial cells (de Pace et al., 2011). Additionally, APEC SEPT362 induces the formation of filopodia and ruffle-like structures on HeLa cells, while cells infected with *hcp* or *clpV* mutants markedly decreased levels of these cytoskeleton rearrangements (de Pace et al., 2010). Interestingly, *hcp* or *clpV* mutants were not involved in intramacrophage replication (de Pace et al., 2010), but a *tssM* mutant showed a reduced intramacrophage survival in J774 macrophages (de Pace et al., 2011).

Also, in the genome of the APEC strain TW-XM, two putative T6SS loci has been found. T6SS1-associated mutants were deficient in adherence to and invasion of several host cell lines, and *in vivo* displayed decreased pathogenicity in duck and mouse infection models; besides decreased bacterial competitive advantage and biofilm formation. In contrast, T6SS2-associated mutants were impaired only in the adherence to and invasion of the mouse BMEC line bEnd.3 and brain tissue of the duck infection model (Ma et al., 2014). These data lead the authors to suggest that T6SS1 could be involved in the proliferation of APEC in systemic infection, whereas VgrG-T6SS2 could be responsible only for cerebral infection.

Recently, a T6SS secreted effector Mn-containing catalase termed KatN was identified in enterohemorrhagic *E. coli* (EHEC). *In vitro*, KatN helps bacterial growth during oxidative stress and is delivered in the host cytosol by the EHEC intramacrophage, resulting in reduced levels of intracellular ROS and greater survival of phagocyted EHEC (Wan et al., 2017).

### Effectors Targeting Bacterial Cells and Antibacterial Interactions

The T6SS direct role for virulence toward eukaryotic cells has been questioned by the discovery that the great majority of T6SSs characterized until now are involved in bacterial growth inhibition (Russell et al., 2012). *E. coli* T6SS gene clusters are classified in three distinct phylogenetic groups, T6SS1 to T6SS3, according to the gene organization and to homologies/similarities. Even though most of the *E. coli* T6SSs, which are until now studied, participate in adherence surfaces (biotic or abiotic), in virulence to several infection models or in bacterial competition, most of the information on *E. coli* T6SSs suggests a role of T6SS1 and T6SS3 for antibacterial activity and of T6SS2 for pathogenesis (Journet and Cascales, 2016). The first T6SS-dependent antagonistic behaviors against neighboring bacteria were shown for T6SS1 and T6SS3 of EAEC 17-2 and T6SS1 of APEC TW-XM (Brunet et al., 2013; Ma et al., 2014; Flaugnatti et al., 2016). Additionally, a role of the T6SS for biofilm formation was shown for T6SS1 of EAEC 17-2 (Aubert et al., 2008) and for T6SS2 of APEC SEPT362 (de Pace et al., 2010).

The antibacterial T6SS function comprises a fascinating offensive and defensive mechanism of the effector–immunity (E-I) pairs (Yang et al., 2018), which are frequently organized in bicistronic units. For now, antibacterial T6SS effectors are divided into effectors for targeting the cell wall, the membrane, and the nucleic acid, as well as other biological functions (Durand et al., 2014; Russell et al., 2014; Alcoforado Diniz and Coulthurst, 2015). When the prey cell is a non-related species/strain, the effectors can be delivered into the cytoplasm or the periplasm of the competitor for degrading the target molecules (Durand et al., 2014). These effectors include amidases, muramidases, and phospholipases that hydrolyze the cell wall (bonds within the peptidic stem, Tae1–4 families, or glucosidic chains, Tge1–3 families, of the peptidoglycan), membrane lipids (ester bonds of phospholipids, Tle1–5 families), and nucleases (DNase activity, Tde) (Journet and Cascales, 2016).

#### Cell Wall Targeting Effectors

Recently, a novel amidase effector in ETEC, called VT1, was identified, and it is encoded within the *vgrG* island (Ma et al., 2018a). VT1 preferentially cleaves the amide bond of D-lactyl-L-Ala crosslinks between N-acetylmuramoyl and L-Ala in peptidoglycan of bacterial cell walls, and play a critical role in the successful establishment of ETEC in host guts. Interestingly, a phylogenetic tree of amidases showed that VT1 is into a separate deep clade and is completely different with identified Tae1–4 amidases. Since the VT1/VTI1 effector/immunity pair is encoded within a typical *vgrG* island, by retrieving these islands in pathogenic *E. coli*, several putative effectors with diverse toxin domains were found. These effectors were designed as VT modules, and among them VT5, an effector widely encoded in ETEC was found to act as a lysozyme-like effector and effectively kills adjacent cells. Bioinformatics prediction showed VT5s share a highly conserved catalytic motif GLxQ with known peptidoglycan glycoside hydrolase of T6SS effector Tge1 (Tse3) (Ma et al., 2018a).

This new retrieval strategy for screening T6SS effectors predicted >200 VT modules from 20 bacterial species and were classified into 11 groups, namely, Tle4, Tle3, Tle1/DUF2235, peptidase, lysozyme-like, two-domain toxin, and other (Ma et al., 2018a).

#### Membrane Targeting Effectors

Tle toxins consist of a large group of enzymes that could be divided into five divergent families bearing phospholipase A1, A2, or D activities. Unlike the Tae and Tge toxins that are antibacterial only, members of the Tle or Tde toxin families target macromolecules present in both eukaryotic and prokaryotic cells (Russell et al., 2013). *E. coli* T6SS1 gene clusters encode putative phospholipases upstream the *vgrG* genes, suggesting their transport by using the VgrG needle as a carrier. These phospholipase genes belong to different families; T6SS1 clusters of AIEC LF82 or UPEC CFT073 carry putative phospholipases of the Tle3 family, while those present on genomes of EAEC 042 and APEC TW-XM are closely related to phospholipases of the Tle1 and Tle4 families, respectively (Journet and Cascales, 2016). Indeed, among the EAEC *sci−1* gene cluster, a gene encoding a putative Tle1 effector followed by a gene encoding a putative lipoprotein is found downstream *vgrG*1. Flaugnatti et al. (2016) found that this Sci−1 T6SS is required for EAEC antibacterial activity and that Tle1 possesses phospholipase A1 (PLA1) and A2 (PLA2) activities, which are responsible for the antibacterial activity of this T6SS. Tle1 is a cargo effector that is delivered into the periplasm of the prey cells using the VgrG1 spike through direct interaction with the VgrG1 C−terminal extension, including a transthyretin-like domain. Autoprotection of the attacker cell is covered through the OM lipoprotein Tli1. Tle1 binds Tli1 in a 1:1 stoichiometric ratio at a nanomolar affinity, and this binding inhibits the phospholipase activity (Flaugnatti et al., 2016).

By using bioinformatics analysis, Russell et al. identified *E. coli* T6SS phospholipase effectors by the existence of a conserved motif, GXSXG, such as *tle1* to *tle4*, but not *tle5* harboring HXKXXXXD motifs (Russell et al., 2013).

#### Nucleic Acid Targeting Effectors

Rearrangement hot spot proteins are filamentous toxins displaying Rhs repeats at N-terminal regions and at highly variable toxin domains at C-terminal regions (Alcoforado Diniz and Coulthurst, 2015). The role of Rhs proteins in bacterial competition is due to the nuclease activity, since these proteins degrade target cell DNA in a contact-dependent manner. RhsI is the immunity protein and specifically neutralizes cognate toxins to protect from autoinhibition (Yang et al., 2018).

Recently, it was found a novel Rhs with an MPTase4 domain (designated as Rhs−CT1) showed an antibacterial effect via T6SS in *E. coli* (Ma et al., 2017b). Rhs-CT modules are widely distributed as T6SS E-I pairs in *E. coli*. These effectors show diverse DNase, RNase, deaminase, and metallopeptidase activities. A total of 10 different toxin domains were found in the Rhs-CTs and were designated as Rhs-CT1 to Rhs-CT10, among them Rhs-CT3 and Rhs-CT4 are putative DNase and Rhs-CT5 to Rhs-CT8 are putative RNase (Ma et al., 2017b). The antibacterial activities of Rhs-CT1, Rhs-CT3, and Rhs-CT5 were experimentally determined. The Rhs-CT3, encoding a C-terminal restriction endonuclease-3 (REase-3) as a member of the DNase clan in the ETEC strain PE027, inhibited recipient cells by more than 100-fold compared with a CT3 mutant and T6SS2 mutant (in ClpV2). On the other hand, the characterization of the Rhs-CT5, which encodes a C-terminal toxin-55 domain with putative RNase activity, showed that O157:H7 cells employ Rhs-CT5 to inhibit other cells and to protect themselves with immunity protein CTI5 (Ma et al., 2017b).

A specific domain architecture retrieval was performed using as a template Rhs-CT1 for searching more analogical target proteins in *E. coli*, and more than 400 Rhs-CT proteins with an N-terminal PAAR motif were found (Ma et al., 2017b).

The VgrG and PAAR proteins have been demonstrated to carry diverse C-terminal extension domains functioning as T6SS antibacterial effectors, while toxic domains fused to Hcp proteins have never been verified to mediate interbacterial antagonism. Ma et al. found, by bioinformatics analyses, Hcp proteins with a C-terminal extension carrying diverse toxin domains, designated as Hcp-ET, which are widespread in Enterobacteriaceae. These genomics analyses identified an extended Hcp with a C-terminal HNH-DNase toxin domain in human O104 STEC strain C227–11 and piglet diarrhea isolate STEC004. This Hcp-ET1 and the T6SS2 cluster are widely prevalent in O104:H4 STEC strains (Ma et al., 2017a). Five of these Hcp-ETs, together with their immunity proteins, were characterized. Hcp-ET1 degrades DNA of the target cell via predicted HNH-DNase activity, and this antibacterial activity in STEC depends on T6SS2-dependent delivery. Hcp-ET2, containing a C-terminal DUF2235 domain in ETEC strain PE321, possesses Tle1 phospholipase activity. The DUF2235 domain was identified as an AB hydrolase1 that acts on lipids and was required for the T6SS-dependent delivery of Hcp-ET2. Hcp-ET3-4 is fused with Pyocin S3 and colicin DNase. Hcp-ET3 with a C-terminal pyocin S3 toxin in ETEC strain PE086 is severely impaired in its capacity to kill *eti3/4*^–^ cells when a deletion of *hcp-et3* in the strain is used. Pyocin S3 is cytotoxic by virtue of its DNase activity. Hcp-ET4 is also functional for interbacterial antagonism via growth inhibition by its colicin DNase toxin (Ma et al., 2017a).

These Hcp-ET toxin domains, such as HNH-DNase, DUF2235, Pyocin S3, and colicin DNase, are widely existent in diverse bacterial species (Ma et al., 2017a).

## *E. coli* T6SS Acquisition

T6SS genes are dispersed in Gram-negative Proteobacteria and highly represented in γ-proteobacteria (Bingle et al., 2008). Thereby, except for *E. coli* B and K-12 laboratory strains, T6SS genes are distributed in most *E. coli* and *Salmonella* species. The genes encoding components and toxins of the T6SS are generally clustered into genetic islands (Bingle et al., 2008; Cascales, 2008). In these regions, the content of G + C is usually more distinct than the core genome, implying these genetic islands were horizontally acquired by gene transfer (Bingle et al., 2008; Cascales, 2008; Boyer et al., 2009). Thus, T6S clusters including the 13 core components, genes for toxins and antitoxins, adaptor proteins that bind both machine components and toxins, and auxiliary proteins required for the assembly of the apparatus or the recruitment and proper delivery of the toxins are usually found within a PAI (Basler, 2015; Journet and Cascales, 2016) as well as on a chromosome with a predisposition to survival or virulence in the host. Thus, they are found in HSI (Hcp-secretion island) of *P. aeruginosa*, *pheU* PAI of EAEC, SCI of *S. typhimurium*, and FPI (*Francisella* PAI) of *F. tularensis*. However, it has been suggested the horizontal transfer of these T6S clusters was not recently acquired since G + C content frequency of clusters is quite similar to other chromosome parts and extensive sequence rearrangements and shuffling has been found (Cascales, 2008). Nevertheless, most of these clusters are located close to rRNA, tRNA, or rearrangement of hot spot (*rhs*) elements, strongly suggesting acquisition by lateral transfer might have occurred in some cases. The *rh*s elements are sequence repetition reservoirs for mediating acquisition of new genetic information or in chromosomal rearrangement (Hill, 1999). Furthermore, due to the multiple copies, it is not clear if T6SS is acquired from gene duplication or different events of genetic transfer.

### T6S Clusters in Pathogenic *E. coli*

*In silico* analysis of T6SS, performed by Shrivastava and Mande (2008), showed a very high frequency of T6S clusters in pathogenic species and a high absence in those non-pathogenic, highlighting functionally this system in conveying pathogenicity to the host. Additionally, it was shown that in most of the sequenced genomes of pathogenic *E. coli* strains, more than 10 T6SS component orthologs exist: in strains EDL933 and Sakai of EHEC (17 orthologs); in strain B171 of EPEC (18 orthologs); in strains 536, UTI189, and CFT073 of UPEC (18, 18, and 15 orthologs); in strain APEC01 of APEC (18 orthologs) with identities ranging from 90 to 99% (Shrivastava and Mande, 2008); as well as in EAEC strain 17-2 and NMEC (neonatal meningitis *E. coli*) strains S88 and IHE3034 (Zhou et al., 2012).

Gene clusters encoding T6SS are categorized in five phylogenetic groups (A–E) according to gene organization and homologies and similarities to TssF core component homologs (similar results are obtained with TssB homologs) (Bingle et al., 2008; Boyer et al., 2009). *E. coli* gene clusters encoding T6SS are categorized in three distinct phylogenetic groups: T6SS1, T6SS2, and T6SS3 into C, D, and B groups, respectively (Journet and Cascales, 2016). Genetic organization of T6SS in *Salmonella*, *Citrobacter*, or *Enterobacter* species is distinct from *E. coli* T6SS1–3 loci. These data suggest these clusters were present in common ancestors or that genetic exchanges occurred between strains that share the same environment. Nonetheless, a common ancestor hypothesis is supported by the fact that each phylogenetic group is found in both intestinal and non-intestinal pathogens such as AIEC, EAEC, EHEC, EPEC and UPEC, APEC, MNEC strains, respectively, and these groups are not found in other bacteria that share similar environments such as *Salmonella* or *Enterobacter* species (Journet and Cascales, 2016).

Among *E. coli* T6SS phylogenetic groups, T6SS1 and T6SS2 gene clusters are the most commonly found in their chromosomes. In APEC genomes for example, the prevalence is T6SS1 (14.6%), T6SS2 (2.4%), and T6SS3 (0.8%). Interestingly, 85% of the T6SS^+^ APEC strains belong to virulent phylogenetic groups (Ma et al., 2013; Wang et al., 2014). Notably, the gene cluster encoding T6SS2 is generally overrepresented in pathogenic bacteria strains with high virulence traits (Journet and Cascales, 2016). An excellent example is seen in EAEC, where T6SS2^+^ strains such as 042 caused diarrhea, whereas T6SS2^–^ strains such as 17-2 and 34b were unable to cause diarrhea in volunteers (Nataro et al., 1995). These data do not necessarily mean T6SSs are directly involved in pathogenesis, but instead, as in other bacteria, T6SSs may prepare the ground for virulence factors by clearing the niche of potential bacterial competitors. For example, in APEC, the T6SS2 has a role on biofilm formation, and a defect in biofilm is accompanied by decreased adherence to epithelial cells (de Pace et al., 2010). It is also likely these phenotypes are the result of impacts in fimbriae gene regulation or alteration in the antibacterial activity due to perturbations of the biofilm structure (Journet and Cascales, 2016). In fact, deletions of T6SS2 genes in APEC SEPT362 affect the expression of type 1 fimbriae and flagella, two extracellular structures required for adhesion and biofilm formation (de Pace et al., 2010, 2011). Regarding potential bacterial competitor clearance, *E. coli* B and K-12 laboratory strains do not harbor T6SS, although gene clusters for T6SS1 and T6SS2 are also found in some non-pathogenic *E. coli* strains such as *E. coli* W (Archer et al., 2011); nevertheless, the T6SS1 cluster in this strain is inactivated by insertion of a mobile element (Archer et al., 2011), and it is not known whether it is functional.

### T6SS Copies and Structural Modules

As mentioned, the core structural components of the T6SS are highly conserved throughout all structural and assembly/disassembly genes, in contrast to the remarkable diversity of T6SS effectors, both on a genetic and functional level (Unterweger et al., 2014). Although there are no studies on *E. coli*, bioinformatics analyses in other bacterial species have shown that structural T6SS components have >95% identity over 37 sequenced strains, while effector module DNA sequences have <30% identity among the same strain set. These genetic differences are increased by the GC content between the core regions and effector modules, in the latter is 6–13% lower than in the structural components (Unterweger et al., 2014). These data indicate that the effector DNA sequences were acquired independently and strongly suggest that effector modules mobilize and are freely exchanged among strains (Kostiuk et al., 2017). In addition, adaptor proteins that mediate biochemical and physical interactions are required for delivering the different effectors (Unterweger et al., 2017). These kinds of proteins have been found also in *E. coli* (Ma et al., 2017b) and are called proteins with domains of unknown function (DUFs) that have been labeled as accessory, adaptor, or chaperone proteins (Unterweger et al., 2017). It has been suggested that the adaptor proteins might influence the effector set of a particular bacterium. The adaptor protein-encoding gene might aid in the acquisition of a new effector protein-encoding gene. The acquisition of effector protein-encoding genes in effector modules may change the effector repertoire of a bacterial strain, which are likely exchanged between strains via horizontal gene transfer (Unterweger et al., 2014, 2017).

The copy multiplicity in *E. coli* T6SS may be associated to various lifestyles and that various T6SS may be coordinated by regulatory mechanisms and the presence of target cells (Bernard et al., 2010). In *E. coli*, the copy number of T6S clusters and their distribution are diverse; APEC and EAEC harbor up to three phylogenetically distinct T6S clusters (Ma et al., 2013): one T6SS gene cluster in UPEC strain CFT037, and in EHEC strains Sakai and EDL933. Even though UPEC and EHEC have an identical number of T6SS gene clusters, they have differences in sequence and organization, suggesting an intricate origin of the T6SS (Wan et al., 2017).

T6SS loci are characterized by the presence of non-core genes inserted in between core elements. Thus, in addition to the principal T6S cluster, other islands encoding Hcp, VgrG, PAAR, and putative toxins could be found disseminated in the genome. Since Hcp and VgrG are carriers for the transport of the effectors, existence of Hcp/VgrG islands suggests these proteins are additional modules that have adapted to the core machine for delivery of specific toxins. In several instances, these small islands are inserted within the core gene cluster. The addition of these distinct modules during evolution may confer specialized functions to these T6SSs. For example, the difference between T6SSs in *Escherichia* and *Salmonella* strains is the nature of the toxin effectors. T6SS1-like clusters generally encode effectors belonging to phospholipases, and T6SS2 clusters have Rhs elements bearing putative activities, whereas *S. enterica* SPI-6 and *Enterobacter cloacae* T6SS gene clusters encode amidases and Rhs-like antibacterial activities (Journet and Cascales, 2016). In addition, by comparing T6SS gene clusters from distinct *E. coli* species, a *vgrG-tle-tli-paar* fragment was found in T6SS1-like operons in the EAEC 042, AIEC LF82, and UPEC UT189 strains. However, they bear differences in the *vgrG* gene and in the E-I pair (Tle1, Tle3, and Tle4 effectors, respectively).

Comparative analysis of genome hybridization revealed gene clusters similar to T6SS in the RDI-1 (RS218-derived genomic island 1) region of the meningitis-causing *E. coli* K1 strain RS2, genes with a similar component to *icmF*, *clpV*, *dotU*, and *hcp2* (Yao et al., 2006). Remarkably, these T6S clusters harbor two genes similar to *hcp* in the chromosome, and they are located next to each other in contrast to other bacterial genomes where *hcp* genes are dispersed (Dudley et al., 2006; Wu et al., 2008). These two proteins similar to *hcp* play divergent roles during the infection by *E. coli* K1, and both participate in *E. coli* K1 pathogenicity in a coordinated way during interaction with HBMECs (Zhou et al., 2012).

T6S apparatus has another level of complexity: VgrG and Hcp comprise essential structural components of the system and also extracellularly shed into the milieu upon T6SS activation; thus, the T6SS includes proteins with a dual-role, structural components/substrates (Silverman et al., 2013). Furthermore, most of the several VgrG and Hcp homologs are linked with only one T6SS; in contrast, most of the 13 core genes exist as a single copy within a specific T6SS locus (Hood et al., 2010; Hachani et al., 2011; Fritsch et al., 2013). These data support the supposition that Hcp and VgrG have a role as adaptor proteins of effector proteins by interacting and recruiting them to the secretion apparatus (Russell et al., 2014).

On the other hand, little information is available regarding the regulatory mechanism of these clusters; whether they are differently regulated or have distinct or similar functions in different conditions is unknown. Taking as an example EAEC 17-2, which encodes two T6SSs in the *pheU* PAI (Dudley et al., 2006), both T6SSs are expressed in different conditions despite both having antibacterial activities (Brunet et al., 2013); the T6SS1 cluster is under the control of the Fur repressor, while the T6SS2 cluster is under the control of AggR. The former is induced during iron starvation, and the latter is a virulence master regulator expressed in the presence of host cells or in synthetic media mimicking the macrophage environment (Dudley et al., 2006). The structure of the T6SS *aai* operon in EAEC suggests gene clusters probably are transcribed by a single promoter as a single unit (Dudley et al., 2006). In another case, the knockout of *hcp1* and *hcp2* genes reduced the ability of the APEC strain CE129 to infect developing chicken embryos. The expression of quorum sensing (QS)-associated genes *luxS*, *lsrR*, and *pfs* were downregulated in the *hcp1* mutant, and the expression of type 1 fimbriae gene *fimA* and the adhesion-related genes *fimC* and *papC* were decreased in the *hcp2* mutant, whereas the expression of antiserum survival factor genes *ompA* and *iss* were inhibited in both *hcp1* and *hcp2* mutants (Ding et al., 2018). Finally, fumarate and nitrate reduction (FNR), a well-known global regulator, was found to regulate expression of the T6SS, affecting the expression of *vgrG* in APEC (Barbieri et al., 2017).

### Alternative T6SS Acquisition

Another source of T6SS variability are so-called orphan immunity genes consisting of open reading frames (ORFs) with considerable homology to immunity genes but are not positioned directly downstream of a cognate effector, however, existing within a given T6SS gene cluster (Kirchberger et al., 2017). These orphan immunity genes are also present in *E. coli* (Ma et al., 2017a). These genes could be acquired by horizontal gene transfer since they are highly similar to those found in the E-I pairs of other strains. It also has been suggested that the successive addition of E-I gene pairs replaces ancestral effectors, yet retains the cognate immunity genes. These data suggest retention of ancient immunity genes might give protection versus neighboring kin bacteria where they were still the old effector. This mechanism, combined with frequent homologous recombination, could be responsible for the high diversity of T6SS E-I genes observed for several bacterial species (Kirchberger et al., 2017).

T6SS-mediated killing causes release of extracellular prey DNA (eDNA), which could then be acquired by the predator strains (Borgeaud et al., 2015) and can recombine anywhere in the genome (Kostiuk et al., 2017). This horizontal gene transfer could result in the acquisition of potentially any gene sequence such as new virulence factors and/or T6SS effector modules. This DNA-mediated fitness advantage is acquired from living cells that were actively killed by the T6SS, not from cells that died as a result of their low fitness (Veening and Blokesch, 2017). It has been observed under laboratory conditions the natural competence following T6SS killing, and it has been shown the effector modules (marked with antibiotic resistance cassettes) were horizontally mobilized and integrated into the genomes. This DNA mobility results in a change in competitive behavior (Thomas et al., 2017). Similar to *V. cholerae*, the T6SS of the naturally competent *Acinetobacter baylyi* ADP1 was shown to promote transfer of a plasmid from prey to predator (Cooper et al., 2017). Interestingly, the horizontal gene transfer efficiency, promoted by the T6SS-mediated lysis of sensitive prey, depends on the mechanism of target cell killing. Overall, potentially all bacteria that encode an antibacterial T6SS and DNA uptake machinery could use their T6SS to acquire new genes (Ringel et al., 2017).

Thus, it is notable that the T6SS of *V. cholerae* is important as a competition factor but also as an enhancer of horizontal gene transfer; in bacteria growth on chitinous surfaces, the competence regulon is expressed, which causes any non-immune neighboring cells to be destroyed, and the released DNA may be a transforming material (Borgeaud et al., 2015). Indeed, recent work has shown that *V. cholerae* experimentally acquires fresh effector genes through horizontal transfer, and these effectors are efficient to kill neighboring cells. Moreover, substitution of parental alleles with novel effectors (one or more) gives the recombinant strain to noticeably outcompete its parent. This work provides strong empirical support to the hypothesis about horizontal exchange of T6SS genes, i.e., by acquiring DNA from killed competitors as well as highlighting the profound impact of horizontal gene transfer in shaping the microbial community structure (Thomas et al., 2017).

## Concluding Remarks

Pathogenic *E. coli* have acquired a variety of sophisticated protein exporting nanomachines to secrete an arsenal of virulence factors implicated in their virulence. Both secretion machineries and effector proteins have been acquired by horizontal gene transfer through several kinds of mobile genetic elements. Some *E. coli* strains harbor multiple distinct T6SS copies, suggesting the multiplicity in T6SS could correspond to various lifestyles of *E. coli*. New T6SS effector genes can be acquired via horizontal gene transfer, e.g., DNA acquired from dead competitors. Substitution of parental alleles (one or more) by new effectors leads the recombinant strain to noticeably surpass the parent. Remarkably, these genes are functionally deployed to compete with neighboring cells, highlighting the profound impact of the genome plasticity in shaping the structure of microbial community.

The field on T6SS has gained much attention in microbiology as an exciting and popular research topic, particularly with *E. coli.* Above 10 known orthologs of T6SS components have been found in a large majority of sequenced genomes of pathogenic *E. coli* such as EHEC strains Sakai and EDL933; EPEC (enteropathogenic *E. coli*) strain B171; EAEC strain 17-2; UPEC strains 536, UTI89, and CFT073; NMEC strains S88 and IHE3034; and APEC strain APEC01 (Shrivastava and Mande, 2008; Lloyd et al., 2009; Moriel et al., 2010). T6SS prevalence in pathogenic *E. coli* strains suggests they may accomplish important functions in virulence. A clear example is the role of injected toxic effectors for acquiring optimal colonization in the host intestine, where high bacterial competition exists for recourses and niches (Ma et al., 2018a). The authors found that ETEC injects a new discovered amidase effector (VT1) that hydrolyzes D-lactyl-L-Ala crosslinks between N-acetylmuramoyl and L-Ala in peptidoglycan. VT1 is encoded together with its immunity protein (VTI1) within a typical *vgrG* island and by searching the *vgrG* islands in pathogenic *E. coli*, a high number of putative effectors that contain diverse toxin domains (designated as VT modules) were found. Among them, a lysozyme-like effector (VT5), widely encoded in ETEC, effectively kills adjacent cells. Thus, VT toxin modules may be critical for pathogenic *E. coli* to seize a significantly competitive advantage by antagonizing and displacing the commensal microbiome to successfully colonize host niches through a T6SS-dependent manner (Ma et al., 2018a).

To date, researchers have only been able to determine their role in biofilm formation, cytoskeleton disruption, and interbacterial competition. However, it is expected that in the coming years, novel functions, other than those associated with host damage and bacterial competition, will be discovered. For example, T6SS has recently been associated to STEC strains with the ability to cause severe hemolytic uremic syndrome (HUS). STEC causes both sporadic infections and outbreaks of enteric disease in humans, with symptoms ranging from asymptomatic carriage to severe HUS. Shiga toxin and the LEE PAI are virulence factors clearly associated to HUS development. However, those factors alone do not exactly distinguish between strains causing HUS and those that do not cause HUS. Surprisingly, the comparison of transcriptomes of *stx2* and *eae*-positive STEC strains (HUS versus non-HUS group) showed 399 of 6,119 gene families were expressed differentially. Moreover, bioinformatic analyses revealed several fimbral operons and putative T6SS were highly expressed in the HUS group in comparison with the non-HUS group, highlighting the relevance of these in the STEC virulence for causing severe disease (Aas et al., 2018).

Given the rapid progress in crystallography, we also anticipate the complete architecture of the T6SS will be defined in the short term. Indeed, during the review process of this paper, a new structure for the membrane complex was published. Initially, a low-resolution negative-stain EM structure of the EAEC membrane complex showed a rotational fivefold symmetry with a stoichiometry of 2:2:2 for TssJ:TssL:TssM. In the new report by using cryo-electron tomography, the fivefold symmetry of the membrane complex was confirmed but *in situ* (immersed into the IM); this approach also identified regions of this structure inserted into the bacterial membrane. Additionally, a high-resolution model from a single-particle cryo-EM revealed five additional TssJ copies producing a stoichiometry of 3:2:2 for TssJ:TssL:TssM, a functionally important periplasm gate constituted by a TssM 11-residue loop that protrudes inside the membrane complex lumen, and finally hinge regions (Rapisarda et al., 2019).

## Author Contributions

All authors listed have made a substantial, direct and intellectual contribution to the work, and approved it for publication.

## Conflict of Interest Statement

The authors declare that the research was conducted in the absence of any commercial or financial relationships that could be construed as a potential conflict of interest.
